# Supplementary material for: Bioaccumulation Behavior and Human Health Risk of Polybrominated Diphenyl Ethers in a Freshwater Food Web of Typical Shallow Lake, Yangtze River Delta
Source: Int J Environ Res Public Health. 2023 Feb 2;20(3):2671. doi: 10.3390/ijerph20032671 (PMC9916311; doi:10.3390/ijerph20032671)
Supplement: Supplementary file 1 [file ijerph-20-02671-s001.zip › ijerph-2063240-supplementary.pdf]

Supplementary Material

Bioaccumulation Behavior and Human Health Risk of Polybrominated Diphenyl Ethers in a Freshwater Food Web of Typical Shallow Lake, Yangtze River Delta

Bei Li [1,2], Juanheng Wang [2,3], Guocheng Hu [1,2,*], Xiaolin Liu [1], Yunjiang Yu [2], Dan Cai [2], Ping Ding [2], Xin Li [2], Lijuan Zhang [2] and Chongdan Xiang [1,2]

[1] South China Institute of Environmental Sciences, Ministry of Ecology and Environment, The Postgraduate Training Base of Jinzhou Medical University, Guangzhou 510530, China
[2] State Environmental Protection Key Laboratory of Environmental Pollution Health Risk Assessment, South China Institute of Environmental Sciences, Ministry of Ecology and Environment, Guangzhou 510530, China
[3] College of Resources and Environment, Yangtze University, Wuhan 430100, China
* Correspondence: huguocheng@scies.org; Tel.: +86-020-29119306

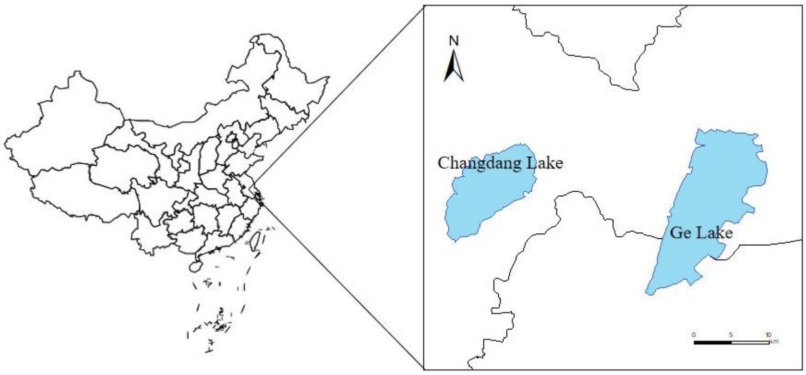

Figure S1. Location of sampling sites: typical shallow lakes in Jiangsu Province, Yangtze River Delta.

Table S1. The basic information of biota samples from typical shallow lakes.

Species	Latin name	Common name	Feeding habitats	Quantity
plankton	*plankton*	Plankton	-	2
Fishes	*Ctenopharyngodon idellus*	Grass crap	herbivorous	3
Fishes	*Mylopharyngodon piceus*	Black carp	herbivorous	2
Fishes	*Parabramis pekinensis*	White amur bream	herbivorous	9
Fishes	*Aristichthys nobilis*	Bighead carp	omnivorous	2
Fishes	*Perca fluviatilis*	English Perch	omnivorous	3
Fishes	*Monopterus albus*	Finless eel	omnivorous	6
Fishes	*Cyprinus carpio*	Common carp	omnivorous	5
Fishes	*Pelteobagrus fulvidraco*	Yellow catfish	omnivorous	12
Fishes	*Hypophthalmichthys molitrix*	Silver carp	omnivorous	4
Fishes	*Hemiculter leucisculus*	White semiknife carp	omnivorous	11
Fishes	*Carassius auratus*	Crucian carp	omnivorous	19
Fishes	*Siniperca chuatsi*	Aucha Perch	carnivorous	2
Fishes	*Coilia nasus*	Coilia Nasus	carnivorous	8
Fishes	*Channa argus*	Northern snakehead	carnivorous	6
Fishes	*Erythroculter ilishaeformis*	Culter alburnus	carnivorous	4
Fishes	*Silurus asotus*	Oriental Sheatfish	carnivorous	6
Fishes	*Sillago sihama*	Silver sillago	carnivorous	3
Fishes	*Anguillar japonica*	Japanese eel	carnivorous	2

Crustaceans	*Eriocheir sinensis*	Crab	omnivorous	12
Crustaceans	*Macrobrachium nipponense*	Shrimp	omnivorous	7
Bottom habitat	*Misgurnus anguillicaudatus*	Loach	omnivorous	13
Bottom habitat	*Cipangopaludina chinensis Gray*	River snail	omnivorous	5

Table S2. The basic information of environmental samples from typical shallow lakes.

Sampling position	Sample types	Sample number	Longitude	Latitude
Ge Lake	Sediment	GL-1	31°38′26.80″	119°50″51.01″
		GL-2	31°36′14.51″	119°48″44.66″
		GL-3	31°33′55.22″	119°47″29.48″
		GL-4	31°30′47.80″	119°45″52.57″
	Surface water	GL	-	-
ChangDangLake	Sediment	CDL-1	31°36′21.06″	119°35″44.03″
		CDL-2	31°36′24.06″	119°35″58.03″
	Surface water	CDL	-	-

S1. ***Experiment analysis***

Instrument analysis

PBDEs were analyzed on an Agilent 7890 gas chromatograph coupled to an Agilent 5795 mass spectrometer using negative chemical ionization (NCI) in selected ion monitoring (SIM) mode, separated by a DB-5HT MS Column capillary (15 m×0.25 mm×0.1 μm, J&W Scientific, Folsom, CA). The operating temperature of the injector was 260 °C. Helium was used as the carrier gas at a constant flow rate of 1.2 mL/min. The temperature program was as follows: 0-5 min, 110 ℃; 5-9.5 min, 200℃; 9.5-14 min, 200 ℃; 14-27.3 min, 300 ℃; 27.3-44.0 min, 300 ℃.

Stable isotope analysis

Homogenized powder samples (0.3 mg) were loaded into tin cups and combusted at 1100 °C for stable nitrogen isotope analysis. The resultant N_2 gas was then analyzed using an isotope ratio mass spectrometer (Delta plus XL, Finnigan) coupled with an elemental analyzer (Flash EA1112, Thermo Fisher). Helium flow: 90~100 mL/min. The isotope ratio was standardized against air using the following formula: $\delta^{15}N=[R_{sample}/(R_{air}-1)] \times 1000‰$, where R is the ratio of $^{15}N/^{14}N$ (Fisk, et al., 2001). For the determination of stable nitrogen isotopes, the measurement error of the analytical method and instrument was ±0.3‰.

Organic carbon analysis

Sediment samples of homogeneous powders (0.2 g) were added to a preweighed glass bottle, and 2 mL of 1 mol/L HCl was added. The samples were left for 18 h and 10 mg was wrapped in tin foil. The total organic carbon (TOC) content in the sediment was determined by an elemental analyzer.

Table S3. Reference dose of pollutants (RfD) and carcinogenic slope factor (CSF) values of the target chemicals.

Contaminant	RfD(mg/kg-day)	CSF (mg/kg-day)$^{-1}$
BDE28	1.0×10^{-4}	-
BDE47	1.0×10^{-4}	-
BDE100	1.0×10^{-4}	-
BDE99	1.0×10^{-4}	-
BDE153	2.0×10^{-4}	-
BDE154	2.0×10^{-4}	-
BDE183	3.0×10^{-4}	-
BDE209	7.0×10^{-3}	7.0×10^{-4}

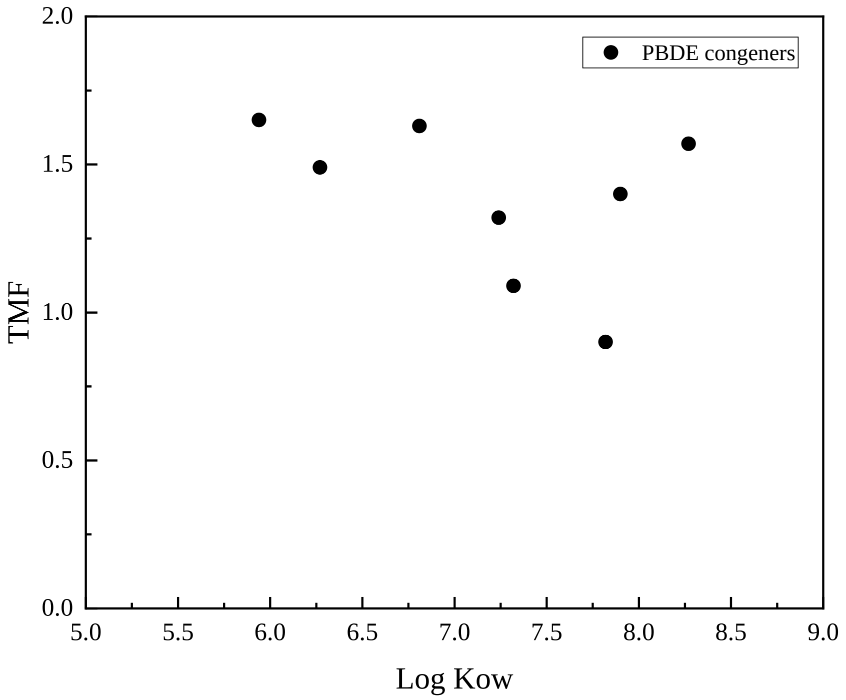

Figure S2. Relationship between TMF values for PBDE congeners and log Kow.

Table S4. The estimated daily intake (EDI) of PBDE congeners via aquatic products consumption for adult residents.

sample	BDE-28	BDE-47	BDE-100	BDE-99	BDE-153	BDE-154	BDE-183	BDE-209	ΣPBDE
Grass crap	0.002	0.006	0.002	0.001	0.019	0.007	0.011	0.006	0.053
Black carp	0.008	0.015	0.005	0.003	0.005	0.008	0.014	0.013	0.071
White amur bream	0.001	0.003	0.002	0.001	0.017	0.006	0.010	0.007	0.047
Bighead carp	0.001	0.005	0.003	0.001	0.016	0.007	0.005	0.010	0.048
English perch	0.008	0.016	0.003	0.002	0.004	0.008	0.004	0.019	0.064
Finless eel	0.010	0.018	0.007	0.013	0.018	0.022	0.011	0.006	0.104
Common carp	0.008	0.054	0.019	0.005	0.026	0.042	0.006	0.019	0.178
Yellow catfish	0.004	0.020	0.005	0.005	0.024	0.017	0.014	0.034	0.123
Silver carp	0.005	0.018	0.005	0.001	0.007	0.009	0.012	0.011	0.067
White semiknife carp	0.004	0.011	0.004	0.001	0.010	0.009	0.011	0.007	0.057
Crucian carp	0.005	0.010	0.003	0.001	0.006	0.007	0.006	0.027	0.065
Loach	0.004	0.011	0.003	0.001	0.008	0.008	0.011	0.017	0.064
Crab	0.004	0.006	0.002	0.001	0.019	0.007	0.011	0.027	0.076
Shrimp	0.006	0.004	0.001	0.001	0.017	0.007	0.013	0.015	0.064
River snail	0.005	0.017	0.021	0.005	0.008	0.020	0.011	0.083	0.170
Aucha perch	0.004	0.030	0.006	0.001	0.009	0.009	0.011	0.027	0.098
Coilia nasus	0.015	0.039	0.010	0.003	0.015	0.011	0.004	0.012	0.109
Northern snakehead	0.002	0.008	0.003	0.002	0.018	0.007	0.005	0.039	0.084

Culter alburnus	0.008	0.024	0.001	0.001	0.020	0.017	0.011	0.019	0.101
Oriental sheatfish	0.015	0.077	0.006	0.006	0.023	0.014	0.012	0.013	0.166
Silver sillago	0.009	0.007	0.021	0.001	0.021	0.007	0.004	0.009	0.079
Japanese eel	0.003	0.016	0.005	0.001	0.016	0.007	0.011	0.007	0.065

Fisk, A. T., K. A. Hobson, and R. J. Norstrom, 2001. Influence of chemical and biological factors on trophic transfer of persistent organic pollutants in the northwater polynya marine food web (vol 35, pg 732, 2001). Environmental Science & Technology 35:1700-1700.
